# Enduring Outcomes of Family-focused Youth Care: A Systematic Review of Child, Parent and Family Functioning After Care and at Follow-up

**DOI:** 10.1007/s10566-024-09837-1

**Published:** 2024-11-19

**Authors:** Bernadette M. Janssen, Jolanda J. P. Mathijssen, Hedwig J. A. Van Bakel

**Affiliations:** 1 TRANZO - Scientific Center for Care and Wellbeing, Tilburg University, PO BOX 90153, 5000 LE Tilburg, The Netherlands; 2 Sterk Huis, PO Box 10139, 5000 JC Tilburg, The Netherlands

**Keywords:** Systematic review, Outcome youth care, Systemic interventions, Short and longer term effects, Child and family functioning

## Abstract

**Background:**

Given the impact of growing up in vulnerable families on opportunities in life and the large numbers of families with severe parenting and child functioning problems who repeatedly receive some form of youth care, it is important to investigate the long-term outcomes of the provided care.

**Objective:**

This study aimed to investigate whether outcomes endure over a longer period of time, by exploring the effects of family-focused youth care on child, parental and family functioning at the end of care and at follow-up moments.

**Method:**

A systematic search was conducted in three databases (Psycinfo, Web of Science and ERIC), using search terms matching youth care and long-term effects. Effects between start and end of care, between end and follow up moments and between start and follow up moments on child, parent and family functioning were systematically mapped out.

**Results:**

Twenty-six articles were included describing effects of family-focused youth care at post treatment and at least one follow-up moment. The results demonstrated positive outcomes in short and longer term as improvement was found in child, parent and family functioning at the end of care and follow-up moments.

**Conclusions:**

Despite the positive outcomes, determining long-term effects of youth care turned out to be difficult, as most studies organize follow-up moments within one year of the end of care. Moreover, there may also be an intertwined effect of the provided and possible follow-up care. More longitudinal research with follow-up moments over multiple years is required to investigate the durability of outcomes.

## Introduction

Although the availability of internationally comparable figures on the number of children in youth care is very limited (Berg-le Clercq et al., 2012), the figures that do exist indicate that this number has increased tremendously since the year 2000 (Lempinen et al., 2019; Olfson et al., 2015; Van Yperen, Van de Maat & Prakken, 2020). In addition, re-referral after youth care trajectories have been completed is a concern, since studies have shown that approximately 30 to 40 percent of cases were re-referred to youth care (Connell et al., 2007; Forrester, 2007). In the longitudinal study by Damen et al. (2021), 94% of families used some form of care in the follow-up period. In view of these increasing numbers, it is relevant to understand the effectiveness of youth care and the durability of these effects.

In general, families referred to youth care are vulnerable families whose core problems generally include severe parenting problems and/or behavioral problems exhibited by the child (Bodden & Deković, 2016; Van Assen et al., 2020; Visscher et al., 2020b) Additionally, these families tend to be confronted with multiple stressors, including marital problems, health problems, social network problems and problems in the domain of justice (Bodden & Deković, 2016; Morris, 2013; Tausendfreund et al., 2014; Veerman, 2015). They frequently also face problems such as low socio-economic status, financial and housing problems or chronic poverty (Bachler et al., 2020; Sousa & Rodriguez, 2012; Veerman, 2015). Since children growing up in these vulnerable families are at an increased risk for poor mental and physical health and developmental problems in many areas of life (Bauman et al., 2006; Pedersen et al., 2019; Van Assen et al., 2020), the durability of youth care is important with a view to increasing these children’s developmental opportunities.

There are different forms of youth care, such as voluntary or mandatory care, home based or (semi-)residential, open or secure stay. Youth care also serves different target groups, e.g. children from families with multiple problems, parents with severe parenting problems, children with mental health or psychiatric problems, and children with severe behavioral and emotional problems. Moreover, approaches in youth care can be roughly classified as either child focused or family-focused. Family-focused youth care implies involvement of parents and other family members in care, which means that not only the child but also parents and other family members are part of the treatment. This approach is based on the system theory, which considers the individual in relation to his environment. In the context of youth care this means that children's problems are viewed in their social context, including the family (Willemse, 2015). Family-focused youth care correlates with better and more consistent outcomes than child-focused care (e.g. Carr, 2019; Sunseri, 2020; Tang, De Haan, Kuiper & Harder, 2023). Based on this premise, this review includes the wide variety of child- and family problems but concentrates on family-focused youth care.

An important goal of family-focused youth care is to improve child functioning by reducing the child’s behavioral and emotional problems. Moreover, it is aimed to improve parenting skills and parental and family functioning through parenting support, enhancing communication between family members, and improving parental functioning by reducing parenting stress and activating their social network (Evenboer, Reijneveld & Jansen et al., 2018; Knot-Dickscheit, 2006; Visscher et al., 2020b). In addition, youth care aims to increase the self-sufficiency of families, avoiding new problems and the need for help (Damen et al., 2021; Nelson et al., 2009; Palmer-House, 2008).

So far, most research on the effects of family-focused youth care have focused on short-term outcomes, with measurements at the start and directly at the end of a youth care trajectory. Generally, meta-analyses and review studies have found positive short-term outcomes on child functioning. Specifically, improvements are found in the child’s wellbeing, their social functioning in peer relationships, and in terms of a decrease in behavioral and emotional problems. Furthermore, improvements in family functioning and family relations have also been found (e.g. Curtis et al., 2004; Pederson et al., 2019; Van Assen et al., 2020). However, as post-treatment measurements generally coincide with the end of care, this type of assessment does not provide information on the durability of the effects of care (Rith-Najarian et al., 2019).

To know whether care has a long-lasting effect, it is important to study whether changes endure over a longer period of time. Based on family-focused youth care’s aim to make families stronger and increase their self-sufficiency (Damen et al., 2021; Hiemstra & Van Yperen, 2015; Palmer-House, 2008), lasting effects of family-focused youth care could be expected. Findings that parental and family involvement in youth care have a positive effect on child-focused outcomes could also result in a durable effect, specifically on child functioning (Curtis et al., 2004; Frensch & Cameron, 2002). By contrast, the vulnerability of families due to often chronic problems in multiple areas of life may undermine durable effects (Forrester, 2007). To learn more about the durability of family-focused youth care effects, the present study systematically reviews longitudinal studies on the short and long-term outcomes of family-focused youth care in terms of child functioning, parenting skills and parental and family functioning. Three main questions were formulated:

1. What are the short-term effects of family-focused youth care in terms of child functioning, parent functioning, parenting skills and family functioning, measured between start and end of care?
2. To what extent do improvements persist, measured during follow-up moments after the end of the family-focused youth care trajectory?
3. Do children and their families function significantly better at follow-up moments than at the start of care?

## Method

This systematic review followed the PRISMA key steps, which describe a guideline for conducting and reporting a systematic literature review (Pati & Lorusso, 2018). These key steps are sequential, including the formulation of the research question, formation of the study team, identification of search domains and publication sources, performing the search, analysis and interpretation, and reporting in a systematic manner. The research team (i.e. the three authors of this article) formulated the research question and the search strategy including inclusion and exclusion criteria. Two researchers of the team conducted the search, analysis and interpretation and started reporting. The third researcher monitored and supervised the process. The followed steps are described below. The authors declare that they have no competing financial interests or personal relationships that could have influenced the work reported in this paper.

### Inclusion and Exclusion Criteria

To ensure all included articles evaluated similar outcomes, the used inclusion and exclusion criteria were based on the target group, intervention form, study design and outcome measures. In addition, inclusion and exclusion criteria were applied regarding publication type and language (see Table 1). For child functioning several types of functioning were included, e.g. behavioral and emotional functioning, manifestations of psychiatric problems, school attendance and drug or alcohol use. Excluded were outcomes that may be a consequence of a youth's behavior but are dependent on or influenced by multiple factors, such as out of home placement and police contacts. Specific inclusion and exclusion criteria are presented in Table 1.

**Table 1:** In- and exclusion criteria for screening for eligibility

Category	Inclusion criteria	Exclusion criteria
1. Research target group	- Families with multiple or complex problems including children with behavioral or emotional problems - Families with severe parenting problems - Children with mental health or psychiatric problems - Children with severe behavioral or emotional problems, including substance abuse and delinquency	- Children with physical of severe intellectual problems, disabilities or disorders as a core problem
2. Intervention form	- Systemic or family focused forms of youth care, both home based and (semi-)residential - Crisis care if systemic or family focused - Juvenile justice facilities if systemic or family focused	- Child focused forms of youth care without any involvement of parents, family or other network - Forms of youth care in which involving parents or family is not standard practice - Long term foster care
3. Study design	- All quantitative studies with pre-post-follow up measurements	- Studies with repeated measurements where it was not clear whether measurements were during, at the end or after treatment - Measurements which started after ending treatment - Qualitative outcome studies - Reviews and meta-analyses - Study protocol
4. Outcome measures	- Child functioning - Parent functioning - Parental skills - Family functioning	- Preventing out of home placement - Judicial outcome
5. Publication type	- Scientific peer-reviewed articles	- Book chapters - Conference abstracts - Comments - Dissertations
6. Form and structure elements - Cultural and linguistic scope	- Written in or translated to English or Dutch - Studies can be conducted in any country	Other languages than Dutch or English
7. Other		- Non usable data - Use of same respondent groups in two or more articles

### Selection of Studies

To answer the research questions, two different search strings were set up. The first search string focused on broad search terms of family-focused youth care, while the second search was performed using specific intervention names. The terms in the first search string contained “youth care” OR “family care” OR “family treatment” OR “home based treatment” OR “outpatient youth care” OR “home based care” OR “residential care” OR “residential youth care” OR “out-of-home-care” AND effect* OR efficacy OR outcome OR evaluat* AND “follow up” OR longitud* OR “long term”.

The second search string contained the following terms: “multisystemic therapy” OR “intensive family treatment” OR “functional family therapy” OR “multisystemic treatment” AND effect* OR efficacy OR outcome OR evaluat* AND “follow up” OR longitud* OR “long term”.

**Fig. 1:**
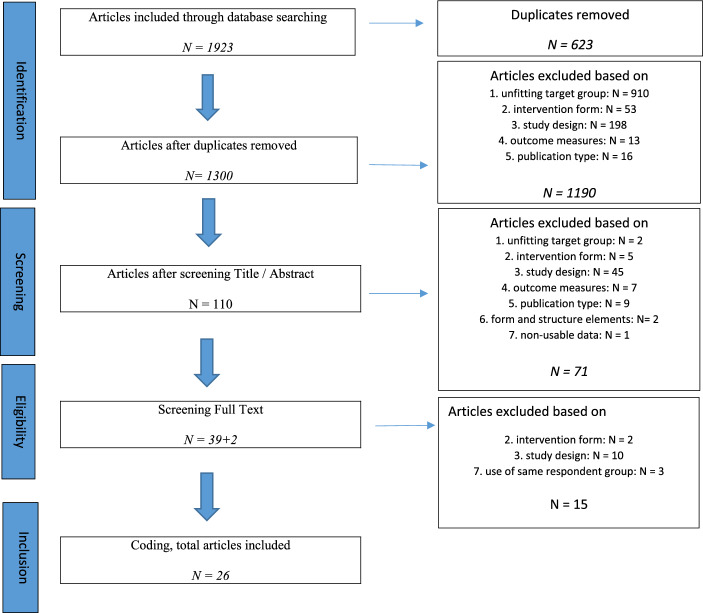
Flow diagram of inclusion of studies

A 5-step procedure was followed to include all relevant studies *(*Fig. 1*).* First, both search strings were set up in databases PsycINFO, ERIC and Web of Science (WOS) in May and June 2023. The search was limited to articles published after 1 January 2000. A total number of 1923 articles was collected: the search with the first string collected 1726 articles, and the search with the second string resulted in 197 articles. The second step was to remove duplicate articles (623 articles), leaving 1300 articles to study closely.

In the third step, the first author screened title and abstract of these 1300 articles to determine which articles matched the inclusion criteria. In order to comply with the interrater reliability and reduce the risk of bias, the second author independently screened 25% of these. In case of doubt whether an article should be included, both authors considered the inclusion and exclusion criteria together in order to reach consensus. Most excluded articles were rejected because of target group or study design. After this screening, 110 articles remained that met the inclusion criteria.

Fourth, the first author screened the full text of all 110 articles for eligibility, while the second author did an independent 25% screening for the interrater reliability. Again, in case of doubt, both authors discussed the target group, method and outcome variables in the text in order to include or exclude the article. From those 110 articles, 39 articles met the inclusion criteria and 71 articles were rejected. Two more articles were found by snowball method, bringing the total number of articles included to 41.

In the final step, the authors set up a coding table, describing the following elements: the number of families or children, average age and sex of the participating children, measuring moments, interventions and intervention duration, outcome category, used instruments, and results at start and end of care and at follow-up moments. If an article did not mention specific outcome data, this was indicated by ‘no specific data available’ (see Table 3). The first author coded all articles, and all authors discussed all elements with doubts about coding in order to reach consensus per coding element. Of the 41 articles, 15 articles were rejected, of which ten because of the study design, two because of the intervention form and three articles were excluded because the same respondent group was used, i.e., the same data, with no specifically other instruments applied.

## Results

### Study Characteristics

Study characteristics are presented in Table 2.

**Table 2:** Study characteristics, intervention name and intervention duration

	Author	Pub Year	Country of data collection	Sample N	Age child (start)	Sex child	Intervention	Intervention duration
	*1st author’s name*			*N start (N last follow up)*	*in years*			*in months (M* = *mean)*
1	Asscher et al	2014	Netherlands	147 juveniles and their parents (111)	M = 16,02; SD = 1.31	27%F, 73%M	MST (multi-systemic therapy)	M = 5.7 months
2	Asarnow et al	2017	USA	42 youths and their parents (42)	M = 14,62; SD unknown	88%F, 12%M	CBFT (Cognitive-Behavioral Family Treatment)	3 months
3	Bachler et al	2020	Austria / Germany	84 families (48)	M = 10,65; SD = 3,52	35%F, 65%M	OFT (Outpatient Family Therapy)	M = 28,8 months
4	Blankestein et al	2018	Netherlands	128 families (87)	MST M = 14,90; SD = 1,38	MST: 36%F, 64%M	MST	MST: M = 4,4 months MST-ID: M = 5,1 months
5	Curtis et al	2009	New Zealand	65 youth and their families (62)	M = 13,85; SD = 1,99	29%F, 71%M	MST	M = 5,1 months
6	Damen et al	2021	Netherlands	275 families (93)	n/a	n/a	IFT (Intensive Family Treatment)	6–9 months
7	Gan et al	2021	Singapore	63 youth and their families (37)	M = 16,3; SD = 1,28	9%F, 91%M	FFT (Functional Family Therapy)	M = 4,7 months
8	Hartnett et al	2016	Ireland	42 families (24)	M = 14,22; SD = 1,45	36%F, 64%M	FFT	3–4 months
9	Henggeler et al	2003	USA	79 families (75)	M = 12,9; SD = 2,1	35%F, 65%M	MST	3–6 months
10	Heywood et al	2016	New Zealand	59 young persons and their families (47)	M = 13,7; SD unknown	30%F; 70%M	FFT	2–4 months
11	Huey et al	2004	USA	79 families (75)	M = 12,9; SD = 2,1	35%F, 65%M	MST	3–6 months
12	Nelson et al	2009	USA	81 children (T3 = 33, T4 = 0)	M = 7,5; SD = 1,1	15%F; 85%M (S)	MST	3–5 months
13	Nuntavisit & Porter	2022	Australia	193 families (193)	M = 13,7; SD1,40	27%F, 73%M	MST	4–5 months
14	Peris & Piacentini	2013	USA	20 youth (16)	M = 12,35; SD = 2,58	45%F, 55%M	PFIT (Positive Family Interaction Therapy)	3–4 months
15	Porter et al	2016	Australia	330 families (153)	M = 13,6; SD unknown	29%F; 71%M	MST	4–6 months
16	Rohde et al	2014	USA	170 adolescents (140)	M = 16; SD unknown	22%F, 78%M	FFT + CWD (Coping With Depression course)	4–5 months
17	Rinne et al	2021	USA	58 adolescents (35)	M = 15,43; SD = 1,68	43%F, 57%M	FFT + EC (enhanced care)	max. 18 sessions (number of months unknown)
18	Rosa-Alcazar et al	2019	Spain	44 children and their parents (44)	M = 6,66; SD = 0,72	25%F, 75%M	CBFT	3 months
19	Salari et al	2014	Australia	29 families (17)	M = 12,92; SD = 1,18	30%F, 70%M	Standard Teen TripleP (Positive Parenting Program)	10 sessions (number of months unknown)
20	Schmidt et al	2006	Germany	70 children (59)	M = 10,9; SD = 3,0	34%F, 66%M	Home treatment program	M = 3,5 months
21	Thogersen et al	2022	Norway	159 adolescents (unknown)	M = 14,7; SD = 1,47	46%F, 54%M	FFT	3—6 months
22	Timmons-Mitchell et al	2006	USA	48 youth (unknown)	M = 15,1; SD = 1,25	22%F, 78%M	MST	M = 4,7 months
23	Tompson et al	2017	USA	3 children and their families (3)	M = 9; SD n/a	67%F, 33%M	FFT-CD (Family Focused Treatment – Childhood Depression)	16 sessions (number of months unknown)
24	Waters et al	2001	Australia	10 children (7)	M unknown, range 10–14 years	40%F, 60%M	CBFT	3–4 months
25	Wijana et al	2018	Sweden	54 adolescents (26)	M = 14,6; SD = 1,3	86%F, 14%M	ICT (Intensive Contextual Treatment for Self-Harm)	3 months
26	Yee et al	2009	USA	77 families with their children (44–57; varies per assessment)	M = 11,8; SD = 3,3	35%F; 65%M	I-FAST (Integrated Family and Systems Treatment)	3 months

#### Participants

All included studies involved children and their families in family-focused youth care: children with behavioral or emotional problems or (psychiatric) disorders, severe parenting problems, families with multiple problems or families having children with significant and long-lasting behavioral problems. A total number of 2,398 families were included in this systematic review. In seven of the 26 studies described, more than 100 families were included at start of care. In 19 of the 26, fewer than 100 families were included, with eight studies including fewer than 50 families.

Most studies focused on children in (pre-) adolescence. In 22 of the 26 studies, children were older than 10 years of age. In three studies, children were 9 years or younger. The study of Damen et al. (2021) did not mention any age of children as this study focused specifically on participating families.

#### Sex Differences

Boys were more represented in the studies than girls. In 17 of the 26 studies the ratio was about 30% girls and 70% boys. In two studies (Asarnow et al., 2017; Wijana et al., 2018) girls were substantially more represented: about 90% were girls and 10% were boys. These two studies both focused on internalizing problem behavior.

#### Type of Treatment

While searching for studies to include in this systematic review, both home based and residential care were of interest. However, after the selection of studies and coding, no studies on short and long term effects of residential care remained based on the inclusion criteria of our review. This means that all included studies were based on ambulatory, home-based care.

#### Interventions

The most commonly used intervention form in the 26 included studies is Multi-Systemic Therapy (MST), followed by Family Functional Therapy (FFT), nine and six times respectively. Cognitive Behavioral Family Treatment (CBFT) was used in three studies. The other studies used other interventions and names (e.g. Intensive Family Treatment (IFT) or Integrated Family and Systems Treatment (I-FAST)), but all with family-focused elements.

#### Intervention Duration

In 21 studies, the duration of the intervention was six months or shorter. The intervention duration in the study of Damen et al. (2021) was described as six to nine months. In three studies, the number of sessions was named but not the exact intervention duration. Only one study (Bachler et al., 2020) reported a much longer duration with a mean duration of 28.8 months.

#### Follow-up Measurements

In 16 studies there was one follow-up measurement after ending care, in nine studies there were two follow-up moments. One study set up three follow-up moments (Rinne et al., 2021). The period of follow-up varied: the shortest was 3 months after ending care (in five studies), and the longest was 3 years after ending care (Bachler et al., 2020). Most of the studies conducted the follow-up measurements in the first year after ending care, in four studies the follow-up moments were over 12 months after ending care (e.g. 18 months, 2 years, 3 years and on average 2.8 years).

#### Measurement of Outcomes

All 26 studies reported on child outcomes; the other concepts (parenting skills, parent functioning and family functioning) were not part of the standard variables measured. Parenting skills (e.g. monitoring and supervision, parenting styles, parental efficacy) were measured in eight studies. Seven out of 26 studies monitored some form of wellbeing of the parent (parent functioning): parental stress, empowerment or health. Family functioning was besides child functioning measured most, in thirteen studies, involving concepts like family adversity, relational functioning and cohesion. Nine studies reported exclusively on child functioning.

#### Measurement Instruments

Most studies used the Child Behavior Checklist (Achenbach, 1991a) and the Strengths and Difficulties Questionnaire (Goodman, 2001; Van Widenfelt et al., 2003) to assess child functioning, besides more specific instruments (e.g. trauma or mood instruments). Various instruments were used for the other concepts.

### Study Findings

Outcomes of family-focused youth care were measured by assessing differences between start and end of care (pre-post treatment), between post treatment and follow-up moments and between start of care and follow-up moments.

#### Differences Between Pre- and Post-Treatment

In 24 out of 26 studies, family-focused youth care led to a significant improvement of child functioning and a decrease in behavioral or emotional problems. Parenting skills showed significant improvements in five out of seven studies where this was measured, and parent functioning significantly improved in four out of six studies. In the other studies, there was improvement without significance, no change was reported or no specific data were available. In 9 out of 13 studies, parents or adolescents reported significant improvements in family functioning at the end of care. In two of the other studies, there were improvements without significance, in one study no changes were found (positive or negative), and one study did measure family functioning pre-test but did not report any outcome data.

#### Differences Between Post-Treatment and Follow-up Moments

Mostly, achieved improvements at end of treatment were maintained or showed further improvement at follow-up measurements on all measured outcome concepts. With some exceptions: on the concept of child functioning, Curtis et al. (2009) reported a significant decrease in school attendance at follow-up. Damen et al. (2021) reported a significant decrease in parental empowerment at follow-up. Porter et al. (2016) showed a significant increase in parental anxiety and depression at 6 months follow-up, but no significant change in parental anxiety and depression at 12 months follow-up as compared to post-treatment.

#### Differences Between Start of Care and Follow-up Moments

In general, improvement is found, and mostly significant, in child, parent and family functioning between start of care and follow-up moments. In 21 out of 26 studies, a significant change was found in child functioning. Parenting skills significantly improved in six out of eight studies between start and follow-up. The least significant improvement between start and follow up was found in parent functioning where significant changes were reported in four out of seven studies. Three studies reported improvement with unknown significance. In nine out of thirteen studies, family functioning improved significantly. No study reported significant deterioration on any of the outcome measures.

#### Intervention Duration Related to Outcomes

Most interventions have a duration shorter than six months, two interventions have longer durations and for three interventions, the number of sessions was described but the exact duration was not explicated. No clear differences were found in short and longer-term outcomes related to intervention duration. Interventions with a shorter duration do not seem to achieve less significant improvements than interventions with a longer duration.

#### Timing of Follow-up Measurements Related to Outcomes

The timing of follow up measurements varied from three months to three years. No clear differences were found in short and longer-term outcomes related to the timing of follow up moments. Follow-up outcomes did not appear to be worse when the follow-up measurement took place later after end of care.

Table 3 shows an overview of pre—post, post—follow-up and pre—follow-up outcomes.

**Table 3:** Timing of follow-up measurements and overview of pre—post, post—follow-up and pre—follow-up outcomes

Author	Follow up period	Outcome / results pre-post	Outcome / results post—follow up	Outcome / results pre—follow up
*1st author’s name*	*follow up in months/years after post treatment*	*in words*	*in words*	*in words*
Asscher et al	pre, post, follow up 6 months	Child functioning: missing pre-post data: no explicit outcome available	Results that were observed at post treatment were still present at follow up (significance unknown)	parents and adolescents reported decrease externalising problems (significance unknown)
Asarnow et al	pre, post, follow up 6 to 12 months	Child functioning: Number of suicide attempts decreases (significance unknown)	Benefits weakened after treatment ended post-follow up (significance unknown)	no explicit data available
Bachler et al	pre, post, follow up 3 years	Child functioning: significant improvement all subscales CBCL pre-post; significant improvement global functioning pre-post	futher improvement most subscales CBCL at follow up of which subscales attention deficit and agressive behavior are significant, subscale somatic problems significant deteroriation; global functioning further improvement (not significant) at follow up	significant improvement all subscales CBCL pre-follow up except scale somatic problems (improvement but not significant); significant improvement global functioning pre-follow up
		Parent functioning: significant improvement pre-post	further significant improvement at follow up	significant improvement pre-follow up
		Family functioning: significant improvement relational functioning and decrease family adversity pre-post	further significant decrease family adversity and further improvement rational functioning (not significant) at follow up	significant improvement relational functioning and decrease family adversity pre-follow up
Blankestein et al	pre, post, follow up 6 months	Child functioning: significant decrease rule breaking behavior pre-post, increase school/work engagement (not significant). No other data available	Improvement decrease rule breaking behavior maintained at follow up. Decrease school/work engagement (not significant). No other data available	Significant decrease rule breaking behavior, no significant change school/work engagement pre-follow up. No other data available
		Parenting skills: No explicit data available	No explicit data available	No explicit data available
		Parent functioning: No explicit data available	No explicit data available	No explicit data available
		Family functioning: No explicit data available	No explicit data available	No explicit data available
Curtis et al	pre, post, follow-up 6 months and 12 months	Child functioning: Behavioral improvement but no significant change, significant increase school attendance pre-post	Significant decrease school attendance post-follow up. No other data available	No sigificant change school attendance pre-follow up. No other data available
		Parental skills: significant improvement parental supervision and monitoring pre-post	Significant increases in parental monitoring continued at 6 months follow up and maintained at 12-month follow up	Significant increase in parental monitoring pre-follow up
		Family functioning: significant improvements pre-post	Significant increases in family functioning at both 6 and 12 month follow up	Significant increases in family functioning pre-follow up
Damen et al	pre, post, follow up on average 2,8 years	Child functioning: significant improvement pre-post	improvement maintained at follow up (no significant change)	improvement child functioning (significance unknown) pre-follow up
		Parent functioning: significant improvement parental empowerment pre-post	significant decrease empowerment at follow up	improvement empowerment (significance unknown) pre-follow up
Gan et al	pre, post, follow up M = 9,82 months	Child functioning: significant improvement pre-post	Improvement maintained at follow up	Significant improvement pre-follow up
		Family functioning: improvement but no significant change pre-post	Improvement but no significant change post-follow-up	Improvement but no significant change pre-follow up
Hartnett et al	pre, post, follow up 3 months	Child functioning (SDQ): Parents and adolescents reported significant decrease behavioral problems pre-post	improvement maintained at follow up	significant decrease behavioral problems pre-follow up
		Family functioning: parents and adolescents reported significant improvement in family functioning on all subscales	improvement maintained at follow up	significant improvement pre-follow up on all subscales
Henggeler et al	pre, post, follow-up 6 months and 12 months	Child functioning: adolescents reported significant improvement pre-post, parents reported significant improvement externalising problems pre-post and improvement (significance unknown) internalising problems, decrease school attendance (significance unknown) pre-post	Adolescents reported maintained improvement, parents reported further improvement (significance unknown) internalising and externalising problems post-follow up, decrease school attendance (significance unknown) post-follow up	Adolescents and parents reported significant improvement in child functioning except significant decrease school attendance pre-follow up
		Family functioning: Adolescents reported increased structure but no significant change pre-post and decreased cohesion during treatment, parents reported increased structure (significance unknown) and no significant change cohesion pre-post	Adolescents reported increased cohesion (significance unknown) post-follow up, parents reported no significant change post-follow up	Adolescents reported no significant change pre-follow up, parents reported significant increase structure, no significant change cohesion pre-follow up
Heywood et al	pre, post, follow up 12 months	Child functioning: parents reported significant reduction CD/ODD behaviors, teachters reported no significant change CD/ODD behaviors; adolescents reported significant reduction of delinquent behavior, parents reported no significant change delinquent behavior and alcohol and substance use pre-post	no explicit data available	parents reported significant reduction CD/ODD behaviors, teachters reported no significant change CD/ODD behaviors; parents and adolescents reported significant reduction in delinquent behaviors, no significant change in alcohol and substanc use pre-follow up
Huey et al	pre, post, follow-up 12 months	Child functioning: adolescents and parents reported improvement for attempted suicide and decrease depression (significance all unknown), improvement anxiety/depression (significance unknown) pre-post	Adolescents and parents reported maintained improvements (significance unknown) post-follow up	Adolescents and parents reported significant positive changes in course over time for attempted suicide and suicidal ideation, anxiety and depression pre-follow up
		Adolescents and parents reported no significant change in parental control pre-post	Adolescents and parents reported no significant change in parental control post-follow up	Adolescents and parents reported no significant change in course over time in parental control pre-follow up
Nelson et al	pre, post, follow up 1 year and 2 years	Child functioning: significant decrease of problem behavior, significant increase of social skills, no significant change academic competence pre-post	improvements in problem behavior and social skills maintained at follow up (no significant change); no significant change academic competence post-follow up	no explicit data available
Nuntavisit & Porter	pre, post, follow-up 6 months and 12 months	Child functioning: significant improvement in internalising, externalising and total behavior problems pre-post	No explicit data available	Significant improvements in internalising, externalising and total problem behaviours pre-6 month and pre-12 month follow-up
		Parenting skills: significant improvement in parental monitoring and decrease in authoritarian and permissive parenting style pre-post	No explicit data available	Significant improvement in parental monitoring and decrease in authoritarian and permissive parenting style pre-6 month and pre-12 month follow-up
		Parent functioning: significant improvement in parental mental health (decrease in depression, anxiety and stress) pre-post	No explicit data available	Significant improvement in parental mental health (decrease in depression, anxiety and stress) pre-6 month and pre-12 month follow-up
Peris & Piacentini	pre, post, follow-up 3 month	Child functioning: decreased symptoms of OCD and improvement global functioning (significance unknown) pre-post	improvements maintained at follow up	Decreased symptoms of OCD and improvement global functioning (significance unknown) pre-follow up
		Parental Skills: decrease of parental blame (significance unknown) pre-post	improvements maintained at follow up	Positive change in course over time (significance unknown) in parental blame pre-follow up
		Family functioning: little changes in cohesion and conflict (significance unknown)	no specific change post-follow up	Positive changes in course over time (significance unknown) in cohesion and conflict pre-follow up
Porter et al	pre, post, follow up 6 and 12 months	Child functioning: significant improvement all subscales and significant decrease internalising and externalising problems and total problems pre-post	improvement maintained at follow ups (no significant change)	decrease total problems and internalizing and externalizing problems (significance unknown) pre-follow up
		Parental skills: significant increase authoritative parenting, significant decrease authoritarian and permissive parenting pre-post treatment	improvement maintained at follow ups (no significant change); except significant decrease subscale authorative parenting regulation post -12 months follow up and sigificant increase indulgent permissive style post-6 and 12 months follow up	increase authoritative parenting, decrease authoritarian and permissive parenting (significances unknown) pre-follow up
		Parent functioning: significant decrease parental depression, anxiety and stress pre-post	significant increase anxiety and stress post—6 months follow up, no sigificant change parental depression, anxiety and stress post—12 months follow up	decrease parental depression, anxiety and stress (significance unknown) pre-follow up
Rohde et al	pre, mid, post, 6 month and 12 month follow-up	Child functioning: adolescents and parents reported significant decrease depression and drug use over the course of time pre-post	Reductions in depression significantly further improved for the group with Mild Depression Disorder (MDD) and sustained for the group without MDD; druguse significantly increased for MDD-group and maintainted for the non-MDD group post-follow-up	Significant decrease depression for the group without MDD, no significant change for the group with MDD pre-follow up
Rinne et al	pre, post, follow up 6, 12 and 18 months	Child functioning: significant decrease depression in course over time pre-post	no explicit data available	Significant decrease depression in course over time pre-follow up
Rosa-Alcazar et al	pre, post, follow-up 3 month	Child functioning: significant improvement in CY-BOCS obsession and in externalizing and externalizing problems pre-post	Further significant improvement post-follow up	Significant improvement pre-follow up
		Family functioning: significant improvement pre-post	Further significant improvement post-follow up	Significant improvement pre-follow up
Salari et al	pre, post, follow-up 3 month	Child functioning: significant decrease behavioral problems, no significant change in social or emotional problems pre-post	Improvement maintained at follow up	Significant change in course over time on most subscales pre-follow up
		Parenting skills: significant decrease of coercive parenting and significant decrease of conflict over child rearing issues between parents pre-post	Improvement maintained at follow up	Significant change in course over time on most subscales pre-follow up
		Parent functioning: no significant change parent personal adjustment pre-post	No significant changes post-follow up	Significant change in course over time parent anxiety (not stress and depression) pre-follow up
		Family functioning: significant decrease of conflict in adolescent-parent relationship, no significant change parents relationship quality pre-post	Improvement maintained at follow up	Significant change in course over time parent–child relation, no significant change parent relation quality pre-follow up
Schmidt et al	pre, post, follow-up 12 months	Child functioning: significant improvement in psychosocial adjustment, level of functioning in global assessment score, significant decrease of psychiatric symptoms pre-post	Improvements on social functioning and behavioral changes maintained at follow-up. Further improvement psychiatric symptoms and global assessment score post-follow up	Significant improvement in psychosocial adjustment, level of functioning in global assessment score, significant decrease of psychiatric symptoms pre-follow up
Thogersen et al	pre, post, follow-up 12 month	Child functioning: significant improvement on all measures pre-post	Further improvement behavioral problems post-follow up, other gained improvements maintained at follow up	Significant improvement behavioral problems pre-follow up, no explicit data available on other measures
		Family functioning: parents reported significant improvement conflict and cohesion, youth reported significant improvement conflict, no significant change cohesion and parental support pre-post	Parents reported no significant changes in cohesion and conflict, youth reported further improvement conflict, no significant change on cohesion and parental support post-follow up	Youth reported significant improvement in family conflict, no explicit data available on other measures pre-follow up
Timmons-Mitchell et al	pre, post, follow up 6 month	Child functioning: significant improvement on all subscales and total scores of CAFAS pre-post	Improvements on all subscales and total scores maintained at follow up, further significant improvement mood and emotions post-follow up	Significant improvement moods and emotions, no other explicit data available pre-follow up
Tompson et al	pre, post, follow-up 4 month and 9 month	Child functioning: decrease in depressive symptoms and diagnostic remission from their depressive disorders, increase of global functioning scores, decrease of internalizing and externalizing behavior problems pre-post	Improvements in depressive symptoms maintained, further increase global functioning, increase of internalizing and externalizing behavior problems (but remained lower than at pretreatment) post-follow up	Improvement in child functioning pre-follow up
Waters et al	pre, post, follow-up 3 month	Child functioning: decrease obsessive–compulsive behavior and anxiety, no change depression and global assessment pre-post	Results maintained post-follow up	Improvement in obsessive–compulsive behavior and anxiety, no change in depression and global assessment pre-follow up
		Family functioning: significant reductions in family accommodation, no significant change in general functioning pre-post	Improvements maintained, no further significant change post-follow up	No significant change pre-follow up
Wijana et al	pre, post, follow-up 6 months and 12 months	Child functioning: adolescents reported significant reductions zelf-harm, suicide attemps, stress, internalising symptoms and improvement emotion regulation, no significant change externalising symptoms. Parents reported significant change internalising and externalising symptoms	Significant deterioration suicide attempts post-follow-up; improvement in self-harm behavior and internalising and externalising behavior maintained post-follow up	No significant change suicide attemps, significant decrease self-harm behavior, stress and internalizing and externalizing problems, significant improvement emotion regulation pre-follow up
		Parent functioning: parents reported significant reduced levels of stress, mothers reported significant decrease in anxiety and depression, fathers reported significantly increase anxiety and depression pre-post	Improvements in mothers' anxiety and depression were maintained	no explicit data available
		Family functioning: adolescents reported no significant change in perceived criticism from both parents, mothers reported significantly lower criticism towards adolescents and significant reductions in emotional overinvolvement, fathers reported a non-significant improvement in criticism and emotional overinvolvement pre-post	Adolescents reported significant reduction in perceived criticism and blame emotional overinvolvement post-follow-up	Adolescents reported significant decrease in perceived criticism, no significant change in emotional involvement from both parents, parents reported significant decrease of levels of criticism and emotional overinvolvement pre-follow up
Yee et al	pre, post, follow up 6 months	Child functioning: parents, youth and professional reported significant decrease problem severity pre-post; significant improvement child functioning pre-post	improvement maintained at follow up (no significant change)	both significant decrease problem severity and improvement child functioning pre-follow-up
		Parental skills: significant improvement parental competence pre-post	improvement maintained at follow-up (no significant change)	significant improvement parental competence pre-follow-up
		Family Functioning: significant inprovement family cohesion and family adaptability pre-post	improvement maintained at follow up (no significant change)	no significant improvement family cohesion and family adaptability pre-follow-up

## Discussion

In this review, the outcomes of family-focused youth care as revealed by its effects on children and their families at the end of care and follow-up moments were investigated. To this end three research questions were formulated:

1. What are the short-term effects of family-focused youth care in terms of child functioning, parent functioning, parenting skills and family functioning, measured between start and end of care?
2. To what extent do improvements persist, measured during follow-up moments after the end of the family-focused youth care trajectory?
3. Do children and their families function significantly better at follow-up moments than at the start of care?

Family-focused youth care leads to favorable results in child, parent and family functioning. Overall, findings in this review indicate both positive short-term and follow-up effects for most families. Since the included studies mostly showed significant changes on child, parent and family functioning between the start and end of care, short-term effects of youth care were found. At follow-up moments, most of the achieved improvements were maintained or had improved further, compared to measurements at the end of care. Considering changes between start of care and follow-up moments, the findings above logically lead to a positive difference between measurements at start and follow-up moments. In general, the children and their families included in this systematic review function significantly better at follow-up moments than at the start of care. These findings support the hypothesis that, despite the pervasive problems these families often face, family-focused youth care achieves positive outcomes.

### Discussion Points

To significantly increase the developmental opportunities of children in families with complex enduring problems, it is important to better understand the durability of family-focused youth care effects. It is however doubtful whether the outcomes we found can be described as durable, long-term effects. The timing of follow-up moments in the included studies varied from a few months until three years after ending care, but most studies set up follow-up moments within one year after the end of care. In this review, no clear differences were found in short and longer-term outcomes related to the timing of follow up moments. However, in only four studies the follow-up measurements took place more than one year after ending care. Thus, the influence of earlier or later timing of measurement moments on outcomes remains interesting to explore.

Additionally, a review by Frensch and Cameron (2002), on residential treatment showed that the longer the follow-up period, the less convincing the found effects of care are, as they seem to diminish over time. This suggests that either the youth care provided may not be durable, or that the ending of youth care should be arranged with extended child or family support (Knorth et al., 2008). The availability of aftercare may be an important factor in increasing the likelihood of durable effects. Trout et al. (2010) describe the need for aftercare services to support the child’s functioning after residential treatment. Studies by Harder et al. (2011) and James et al. (2013) showed positive effects of aftercare, also as a successor to residential care. The use of aftercare in home-based trajectories appears to be an unexplored theme. Furthermore, as Damen et al. (2021) report, 94% of families in their study were provided some form of care in the follow-up period. This may be aftercare, in which the provided care is continued at a lower frequency, intended to consolidate achieved results, or follow-up care, in which another form of care is offered to help meet the family’s care request. In this light, it is quite possible that families in the studies included in this review also used aftercare, follow-up care, or were re-referred to some form of youth care (Connell et al., 2007; Damen et al., 2021; Forrester, 2007). This could mean that the follow-up measurements in this review did not exclusively map out the follow-up effects of the original care but also the effects of other care received later. This may specifically be the case when follow-up measurement moments are further away from the original care (Rith-Najarian et al., 2019). The positive follow-up effects found in this review may be influenced by this possibility.

Given the different times of follow-up measurements and the possible use of aftercare or follow-up care, it is difficult to draw any firm conclusions about long-term effects. The meaning of the term ‘durability’ is in itself a point of attention, since the terms ‘durability’ or ‘long-term effects’ are not clearly defined (Rith-Najarian et al., 2019).

In addition to the short periods of follow-up measurements, it is notable that the duration of interventions was also often shorter than one year. This raises the question whether it is feasible to achieve durable effects for families with complex problems within a short period of care.

All included studies measured the child’s functioning during and after care. Fewer measurements were performed for the other concepts (i.e. parenting skills, parent functioning and family functioning). As we specifically selected on family-focused youth care, aimed at both children and their families, it is striking that the effects of care on parental and family functioning were not structurally mapped out. Also, the impact of involving the social network and increasing self-sufficiency as part of family functioning was not measured in any of the studies. This is all the more remarkable, since parental and family involvement positively correlate with child-level treatment outcomes (Curtis et al., 2004; Frensch & Cameron, 2002). However, Tang et al., in their review (2024), did find some indications of a positive association between family-focused care and family outcomes of care, but in general there appears to be no clear evidence yet.

Studies of youth care in general often differentiate between home-based and residential care. Assuming that these forms of care support the same target groups from the same family-focused vision, in this review we tried to study all forms of family-focused youth care. However, when screening articles for eligibility, all articles describing outcome studies for residential care were excluded, mostly for reasons of study design (e.g. no follow-up measurements or measurements only started after the end of care) and of intervention form (e.g. child-focused instead of family-focused). As a result, this review inadvertently focuses on home-based family-focused youth care interventions, and the effects of residential family-focused youth care at end and follow-up moments were not captured.

Lastly, the interventions in the included studies involved some form of family treatment and the provided intervention elements were described in most studies. However, the association between these elements and outcomes of care was not described, making it unclear which elements contributed to the improvements. There is now a growing focus on identifying the connections between intervention elements, known effective elements of care and outcomes of care. This is an important theme for further research. (Renmans & Pleguezuelo, 2023).

### Limitations in Study Design

Despite the two search strings used, only a limited sample of articles was suitable. In total, 26 articles met the inclusion criteria. Consequently, the conclusions of this systematic review should be treated with some caution. Due to the larger number of studies measuring child functioning, most of the solid conclusions pertain to the short and longer-term effectiveness for child functioning, and to a lesser extent to parenting skills, parent functioning and family functioning. While the focus of the study was on family-focused youth care for vulnerable families, little longitudinal research turned out to be available with measurements both before and after treatment. Three possible causes could be identified. First, the limited availability of longitudinal research might be due to publication bias, as studies reporting significant effects of youth care may be more likely to be accepted for publication in a journal. Conversely, studies that report no or fewer significant outcome effects may not be published or even offered for publication, so they may be difficult to find (Gutterswijk et al., 2020; Strijbosch et al., 2015). Second, the limited amount of longitudinal research may involve organizational issues, like limited available financial resources and manpower and the challenge of finding clients willing to participate on a long-term basis in the research. Third, in most outcome studies of residential care, measurements were conducted only at start and end of care (e.g. Gevers et al., 2021; Leloux-Opmeer et al., 2018), or the first measurement moments take place after discharge as displayed in the review of Yeheskel et al. (2020). As a result, less research with measurements between start and end of care and at follow-up may be available, leading to less insight into the durable effects of care.

Another limitation is the sample size of studies at start and end of care, resulting in a relatively small power at the different measurement moments in most studies. At start, sample sizes vary from three families (Tompson et al., 2017) to 330 families (Porter & Nuntavisit, 2016). In most studies, there were less than 100 respondents at start of care, in eight of these there were less than fifty. In most studies, except for two (Rosa-Alcazar et al., 2019; Tompson et al., 2017), the number of respondents diminished at follow-up. For example, in the study by Nelson et al. (2009), at the first follow-up moment (1 year after care) the number of families dropped from 81 to 31, and at the second follow-up moment (2 years after care), no participants were left.

Lastly, most included studies did not focus specifically on long-term effects of family-focused youth care but investigated differences in outcomes between two interventions. As a result, specific outcomes at the end of care and follow-up moments were not always explicated, making it complex to filter out the outcomes sought.

### Implications for Further Research

Following the outcomes and limitations of this review, further research into the long-term outcomes of family-focused youth care is recommended. First, more longitudinal research on the durability of family-focused youth care is needed. As the timing of follow-up assessments in the used studies varied widely, one implication is to set up multiple follow-up moments, from shortly after the end of treatment to several years after treatment. In light of the discussion points above, this requires a close monitoring of any aftercare or follow-up care and studying the intertwined effects of original and after-care or follow-up care. It is important to study not only short and long-term changes in child functioning but also, or specifically, changes in parenting skills and parent and family functioning. In addition, the focus should not only be on the significance of changes between measurement moments, but should also map out to what extent respondents report above or below the clinical cut-off score about the child’s, parent’s and family’s functioning at the start and end of care and at follow-up moments. When reported high above this point at start, improvement is more likely to be measured at the end of treatment than when functioning is measured in the non-clinical range at start. Moreover, when a clinical score is measured at start, it is worthwhile mapping out whether the score at the end of care or at follow-up moments is still in clinical range or not. To better understand this, it is important to examine the extent to which changes evolve from clinical to non-clinical functioning between the different measurement moments. In this review, eventually only home based interventions were included. This implies that more longitudinal research with an emphasis on outcomes in family-focused residential care is needed.

Second, no clear differences were found in shorter and longer-term outcomes related to intervention duration and the timing of follow-up measurements. As both the numbers of studies with an intervention duration longer than six months and studies with follow-up measurements over one year after care were limited, it requires further research to investigate possible correlations.

Third, family-focused youth care covers a broad target population (e.g., families with multiple problems, children with severe mental health, behavioral or emotional problems). This review did not differentiate in outcomes between the different target groups. In further research, it would be interesting to zoom in on these differences in relation to short and longer-term outcomes.

Finally, this review study shows that for most families, family-focused youth care leads to significant improvements in their functioning. However, these improvements were not evident in all families. Therefore, it is important to understand what does or does not work well for which families. Questionnaires yield a quantitative overview of whether changes have occurred between the start and end of treatment and follow-up moments. Qualitative research gives a more in-depth insight in how changes were achieved. To investigate *how* and *for whom* youth care works, multi-method research is recommended (Hattum et al., 2019). A combination of quantitative and qualitative research into the effects of youth care as experienced by children and parents and what the effective elements of care are, would contribute to increasing knowledge about what works, for which families, and under what circumstances (Renmans & Pleguezuelo, 2023).

### Conclusion

Despite its limitations, this systematic review provides new insights into the outcomes of youth care. It reveals improvements in child, parental and family functioning at the end of care and follow-up moments. The results also imply several leads for future research. In addition to understanding the improvements, it is important to investigate how these effects are achieved and to identify which efforts contribute to this improvement.
